# Cost-effectiveness of hypofractionated versus conventional fractionated radiotherapy for the treatment of men with early glottic cancer: a study in the Brazilian public and private health system

**DOI:** 10.1186/s12913-023-09397-5

**Published:** 2023-06-08

**Authors:** Marina Lourenção, Gustavo Viani Arruda, Lucas Penna Rocha, Julia Simões Corrêa Galendi, Jorge Caldeira de Oliveira, Alexandre Arthur Jacinto

**Affiliations:** 1 Department of Business Administration, School of Economics, Business Administration and Accounting at Ribeirão Preto, University of São Paulo, Sao Paulo, Brazil; 2 Department of Radiation Oncology, Ribeirão Preto Medical School, University of São Paulo, Sao Paulo, Brazil; 3 Department of Head and Neck, Ribeirão Preto Medical School, University of São Paulo, Sao Paulo, Brazil; 4 Faculty of Medicine and University Hospital of Cologne, Cologne, Germany; 5 Institute of Health Economics and Clinical Epidemiology, University of Cologne, Cologne, Germany; 6 Department of Radiation Oncology, Barretos Cancer Hospital, Sao Paulo, Brazil

**Keywords:** Early glottic cancer, Radiotherapy, Hypofractionation, Conventional fractionation, Cost-effectiveness

## Abstract

**Background:**

This study aims to evaluate whether hypofractionated radiotherapy (HYPOFRT) is a cost-effective strategy than conventional fractionated radiotherapy (CFRT) for early-stage glottic cancer (ESGC) in the Brazilian public and private health systems.

**Methods:**

Adopting the perspective of the Brazilian public and private health system as the payer, a Markov model with a lifetime horizon was built to delineate the health states for a cohort of 65-year-old men after with ESGC treated with either HYPOFRT or CFRT. Probabilities of controlled disease, local failure, distant metastasis, and death and utilities scores were extracted from randomized clinical trials. Costs were based on the public and private health system reimbursement values.

**Results:**

In the base case scenario, for both the public and private health systems, HYPOFRT dominated CFRT, being more effective and less costly, with a negative ICER of R$264.32 per quality-adjusted life-year (QALY) (public health system) and a negative ICER of R$2870.69/ QALY (private health system). The ICER was most sensitive to the probability of local failure, controlled disease, and salvage treatment costs. For the probabilistic sensitivity analysis, the cost-effectiveness acceptability curve indicates that there is a probability of 99.99% of HYPOFRT being cost-effective considering a willingness-to-pay threshold of R$2,000 ($905.39) per QALY (public sector) and willingness-to-pay threshold of R$16,000 ($7243.10) per QALY (private sector). The results were robust in deterministic and probabilistic sensitivity analyses.

**Conclusions:**

Considering a threshold of R$ 40,000 per QALY, HYPOFRT was cost-effective compared to CFRT for ESGC in the Brazilian public health system. The Net Monetary Benefit (NMB) is approximately 2,4 times (public health system) and 5,2 (private health system) higher for HYPOFRT than CFRT, which could open the opportunity of incorporating new technologies.

**Supplementary Information:**

The online version contains supplementary material available at 10.1186/s12913-023-09397-5.

## Background

Radiotherapy is an effective definitive intervention that promises anatomic and functional preservation to patients with early-stage glottic cancer (ESGC) [1–3]. Historically, in most series, a conventional fractionation (CFRT) with a dose per fraction of 1.8–2.0 Gray (Gy) in 33 to 35 daily fractions has been used to treat ESGC [1]. The developed knowledge in radiobiology has sparked interest in alternative fractionation schedules to improve local control [4, 5]. Some researchers have investigated hypofractionation schemes using daily dosing between 2.0 -3 Gy with reduced fractions (20–30 fractions) and the overall treatment time [6, 7]. The initial outcomes of retrospective studies comparing the two schedules demonstrated that the use of CFRT in ESGC can increase the chances of local recurrence compared with HYPOFRT, which can result in more salvage laryngectomies surgeries with a significant impact on the patient's quality of life and the costs for the public health system [7].

In a retrospective comparison that included 145 patients with ESGC [7], the HYPOFRT group had higher loco-regional control in T1N0 patients than the CFRT group (95 vs. 75%, p: 0.002). The 5-year cancer laryngectomy-free survival (LFS) was 95% for the HYPOFRT compared to 75% in the CFRT group (p: 0.003).

This evidence was strengthened by the Japanese landmark randomized controlled trial (RCT) [8], which shows superior local control in ESGC with HYPOFRT with a dose per fraction of 2.25 Gy per day in comparison to CFRT [8]. The study by [8] investigated 180 patients and indicated that the five-year local control rate was 92% for HYPOFRT compared to 77% in the CFRT group (p: 0.004). The corresponding 5-year cause-specific survival rates had no significant difference.

The HYPOFRT schedule is an interesting alternative for upper-middle-income countries with limited Linear Accelerator (LINAC) access. Moreover, HYPOFRT is more convenient for patients and can reduce financial toxicity, avoiding patients traveling long distances several times to receive their treatment [9].

Brazil is a large upper-middle-income country with a shortage of LINACs to treat cancer patients [10, 11]. Larynx cancer is ranked among the top 10 most incident cancer in the Brazilian population, with 6000 cases in 2020[12]. Thus, adopting HYPOFRT to treat laryngeal cancer could represent an opportunity for treating more patients and breaking the access barriers to LINACs.

In Brazil, 80% of the population is covered solely by the public health system of more than 200 million people [13]. The radiotherapy reimbursement in the public system does not specify what radiotherapy technique or fractionation should be used, and it pays a fixed value for the procedure [14]. Consequently, radiation oncologists can choose the schedule and radiotherapy technique to be delivered.

Moreover, in an upper-middle-income country with a restricted budget to be invested in the health system, the resources spent inefficiently can affect all the patient network care [15]. Based on that premise, this present study was designed to answer this question: Is HYPOFRT a cost-effective strategy compared to CFRT for treating men with EGSC in the context of the public and private health system of an upper-middle-income country such as Brazil?

## Methods

A Markov model was designed to simulate the clinical outcome of a 65-year-old man with early-stage glottic cancer (T1-2N0M0). We used the TreeAge Pro software (TreeAge Healthcare Version 2023) to estimate the costs and benefits. The costs were expressed in Brazilian Reais (R$), and the benefits were described as quality-adjusted life-year (QALY) and life years gained (LYG). The software calculates the incremental cost-effectiveness ratio (ICER) by dividing the difference between the two strategies in lifetime costs by the difference in lifetime effects (QALYs and LYG). This study is reported according to the Consolidated Health Economic Evaluation Reporting Standards (CHEERS) checklist [16] (Supplementary Table 1).

The analysis was conducted from the perspective of the public (Brazilian Unified Healthcare System—SUS) and the private health system as the payers. The cycle length was one year, and the time horizon was set to 15 years to capture the lifetime consequences of the ESGC treatment [17], as suggested by other cost-effective studies for cancer treatment [18, 19]. In line with recommendations from the Brazilian guideline for economic evaluations, costs, and benefits were discounted at 5% [20].

When entering the model, the target population was a cohort of 65-year-old men with ESGC, representing the Yamazaki cohort average age used for the transition probabilities calculation. According to Globocan [21], larynx cancer is most common in men older than 60 years. In 2020, men had 6.58 times more new cases of laryngeal cancer than women [21].

### Strategies for comparison and model overview

We have compared two radiation strategies: (1) HYPOFRT, defined as 56.25 Gy in 25 fractions within 5 weeks for a minimal tumor or 63 Gy in 28 fractions within 5.6 weeks for larger than minimal tumors, the radiation fraction size was 2.25 Gy; and (2) CFRT defined as 60 Gy in 30 fractions within 6 weeks for minimal tumors (two-thirds of the vocal cord or less) or 66 Gy in 33 fractions in 6.6 weeks for larger than minimal tumors (more than two-thirds of the vocal cord), the radiation fraction size was 2 Gy.

The Markov model was used because it is suitable for chronic diseases where events are likely to recur over time [17]. A Markov model comprises a finite number of health states where a patient can be found. The patient will have only one health state in a period [17].

Markov model allows us hypothetically simulate cohorts of patients with transitions between different health states using fixed time increments. In other words, this model considers a hypothetical patient with ESGC and simulates the possibility of the occurrence of events after radiotherapy treatment.

**Fig. 1:**
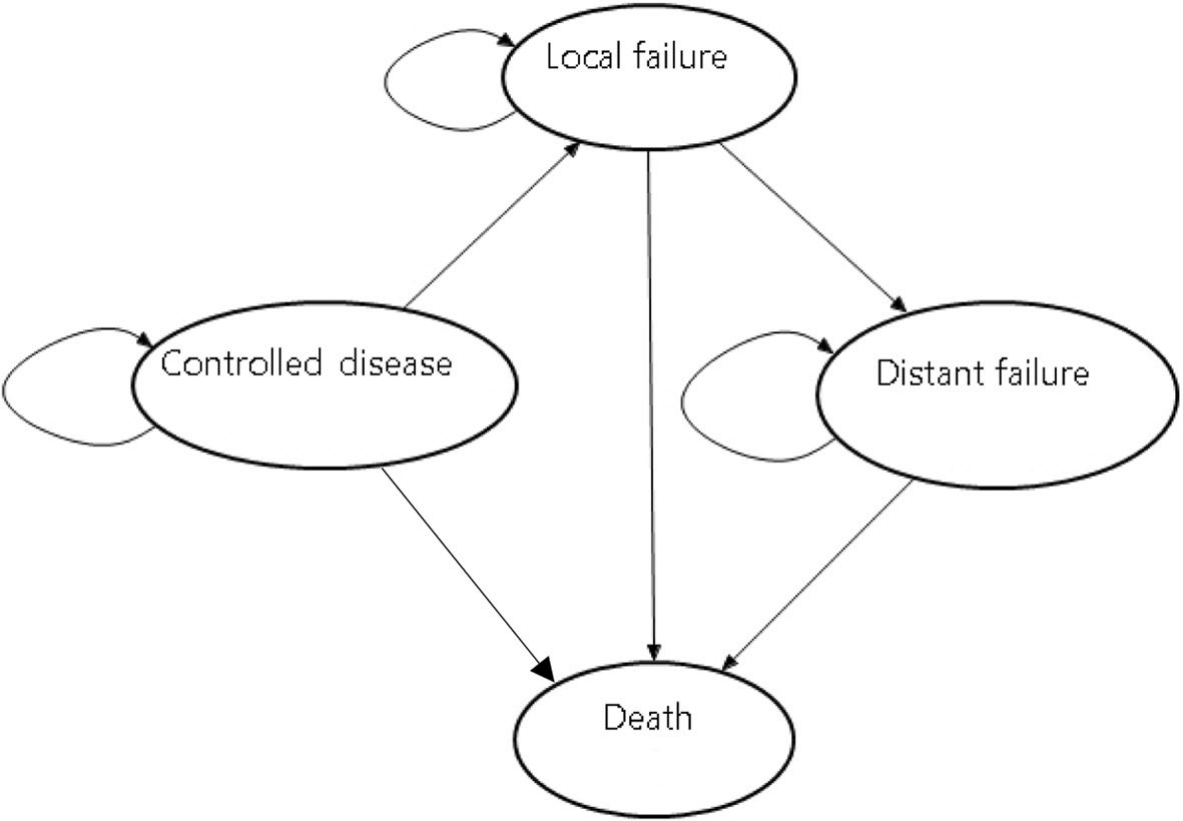
Heath states for Markov model

The Markov model structure was developed based on consultancy with experts and previous models. Over the course, the patient may develop local recurrence, distant recurrence, or death from any causes. Figure 1 describes the health states used in the simulation.

Patients in the model started in the health state 'controlled disease,' i.e., no evidence of disease or radiation collateral effects. These patients could either stay there or move to the state 'local failure.' It was assumed that when patients had a local failure, they were salvaged with total laryngectomy and neck dissection. Thus, patients in the 'local failure' state could either stay there, move to distant failure, or die (absorbing state). The patients that remained in the ‘local failure’ state were considered to have had a local recurrence but were treated; that is, they had disease remission. Patients in the 'distant failure' state could either stay there or die.

### Utilities

The utility is an econometric measure given to a health status ranging from 0 (death) to 1 (perfect health). In the model, each health state, such as controlled disease, local failure, and distant metastasis, was associated with a utility value (Table 1). The utility of controlled disease was estimated using the study by [22], which uses EQ-5D to assess the health-state utility value for head and neck patients after curative radiotherapy without evidence of recurrent disease. The utility value of local failure was estimated using the study by [23], which uses time trade-off (TTO) to assess the utility value of locally advanced laryngeal cancer patients after total laryngectomy. Finally, the utility value of distant failure was evaluated using the study by [24], which uses the EQ-5D-3L to indicate the health state utility value for elderly head and neck patients with distant metastasis. QALYs are a composite measure of quality and quantity of life. To combine utility values, the multiplicative method was used [17].

**Table 1:** Inputs used in the Markov model: probabilities, utilities, and costs

Variable	Value (SD)	Source
***Probabilities***		
HYPOFRT		
From controlled disease to controlled disease (probability of staying in the same health state)	y1:0.97; y2: 0.96; y3: 0.94; y4:0.94; y5:0.93 (0.014)	[8]
CFRT		
From controlled disease to controlled disease (probability of staying in the same health state)	y1:0.94; y2: 0.90; y3: 0.85; y4: 0.81; y5: 0.77 (0.07)	[8]
HYPOFRT or CFRT		
From local failure to distant failure	y1: 0.008; y2: 0.017; y3: 0.026; y4: 0.034; y5: 0.043 (0.013)	[8]
From local failure to death	y1:0.025; y2:0.049; y3:0.073; y4: 0.09; y5: 0.12 (0.037)	[8]
From distant failure to death	y1: 0.601893; y2: 0.84; y3: 0.93; y4: 0.97; y5: 0.99 (0.14)	[8]
From controlled disease to death	0.03 (0.014)	[25]
***Utility***		
Controlled disease	0.85 (0.017)	[22]
Local failure	0.57 (0.060)	[23]
Distant failure	0.42 (0.006)	[24]
***Costs (R$)***		
HYPOFRT_and_CFRT_initial_costs	Public health system: 4,168.00 (1667.2); Private health system: 37,396.24 (14,958.49)	[26, 27]
Controlled disease	Public health system y1: 823.92; y2: 707.16; y3: 220.41; y4: 220.41; y5: 220.41 (301.42) Private health system y1: 14,611.79; y2: 13,919.07; y3: 2,897.68; y4: 2,897.68; y5: 2,897.68 (6231.18) Public health system	[26, 27]
Local failure	y1:1,804.23; y2: 707.16; y3: 220.41; y4: 220.41; y5: 220.41 (687.01) Private health system y1: 20,838.67; y2: 13,919.07; y3: 2,897.68; y4: 2,897.68; y5: 2,897.68 (8300.39) Public health system	[26, 27]
Distant failure	y1: 5,400.76; y2: 484.00; y3: 220.41; y4: 220.41; y5: 220.41 (2371.48) Private health system y1: 24,211.79; y2: 13,919.07; y3: 2,897.68; y4: 2,897.68; y5: 2,897.68 (9573.99)	[26, 27]
***Probabilistic sensitivity analysis***		
Probabilities	Beta distribution	TreeAge
Utility	Beta distribution	TreeAge
Costs	Gamma distribution	TreeAge

### Costs

As suggested by the Brazilian guideline for economic evaluations [20], cost data were expressed in Brazilian currency (Reais). The 2022 unit cost values for the public health system were obtained from the official Brazilian Unified Healthcare System (SUS) database, named the Table of Procedures, Medications and Ortheses, Prostheses, and Special Materials for the National Health System (DATASUS SIGTAP) [26]. The 2022 unit cost values for the private health system were obtained from the table used for the health insurance companies called the Brazilian Hierarchical Classification of Medical Procedures (CBHPM) [27]. The private health system pays higher value than the public health system. Most of the values in CBHPM are more than 200% of the amounts the public health system pays. The public and private health systems pay a fixed value independent of the technique and number of radiotherapy fractions. Resource use (i.e., the quantity regarding each diagnostic exam and clinical procedures during the follow-up) was estimated for each health state based on recommendations from an international guideline (NCCN, 2022), with minor adaptations for Brazilian reality made by two radiation oncologists and one head and neck surgeon. Annual costs were calculated for each Markov model health state. Tunnel states were implemented in order to attribute different annual costs per year per health state. The costs involved with each state are described as follows:

1. Controlled disease state includes costs of follow-up exams and consultations with doctors, dentists, psychologists, nutritionists, and speech therapists.
2. The local failure health state comprises the costs of total laryngectomy with neck dissection and follow-up exams and consultations.
3. The distant failure health state includes costs of adjuvant chemotherapy for head and neck cancer and follow-up exams and consultations.

Table 1 summarizes the costs input data per health state and year. Supplementary Table 2 comprises more detailed costs highlighting all the resources used for diagnostic, clinical, and surgical procedures. To allow comparisons with other settings, conversion of the results presented in Brazilian real (R$) to United States dollar ($) was performed by using a web-based tool (CCEMG – EPPI-Centre Cost Converter) for the year 2022. This tool considers the Gross Domestic Product deflator index and the Purchasing Power Parities for GDP (‘PPP values’) to convert currencies.

### Probabilities

After a systematic literature search on the PubMed database to find randomized clinical trials that compared HYPOFRT and CFRT (search strategies are described in Supplementary Table 3). Two randomized clinical trials compared HYPORFR and CFRT for early-stage glottic cancer [8, 28]. The landmark Japanese trial was chosen to provide the transition probabilities due to the higher sample size [8]. We calculated yearly transition probabilities based on yearly control rates reported by Yamazaki et al. graphics that showed different control rates for each year. The probability of a patient having a local recurrence after the radiotherapy treatment increases yearly over 5 years and was modeled using tunnel states. According to Yamazaki’s study the transition probability remains the same from year 5 onwards. Supplementary Table 4 comprises more detailed probabilities and their values regarding sensitivity analysis. The probabilities high and low values were calculated based on their confidence intervals that was reported at Supplementary Table 2.

### Model validation and sensitivity analysis

To validate the Markov model, we consulted experts (two radiation oncologists and one head and neck surgeon) on the health state conceptual suitability and input data appropriateness. Besides, we also compared our Markov model with previous ones published in the literature to validate the disease process. Few studies present a Markov model to illustrate possible health states for larynx cancer. The study by [29] shows a Markov model for advanced laryngeal cancer and, due to that, does not include the ‘local failure’ state. The study by [30] proposes a Markov model for locally advanced head and neck cancer and presents health states similar to the ones elaborated by the present study.

We conducted a deterministic sensitivity analysis to characterize parameter uncertainty in the outcome measures. We performed deterministic sensitivity analyses by varying probabilities and utilities considering their confidence intervals reported in Supplementary Table 2. The costs varied within 40% to obtain a comprehensive range, as suggested by [31]. We also performed a probabilistic sensitivity analysis with a Monte Carlo simulation with 10.000 simulations. This method contributes to assessing how a simultaneous change of several variables affects the ICER values. Gamma distributions were used for cost parameters. Probabilities and utilities were considered to be beta-distributed.

## Results

### Base-case analysis

From the public health system perspective, the HYPOFRT strategy costs R$7,008.54 ($3172.72), and the CFRT costs R$7,993.29 ($3618.51). Thus CFRT has an incremental cost of R$ 984.74 ($445.79).

When considering the values paid by the Brazilian private health system, the HYPOFRT strategy costs R$77,899.27 ($35,264.50), and the CFRT costs R$88,594.23 ($40,106.03), CFRT has an incremental cost of R$ 10,694.96 ($ 4,841.54).

Accordingly, from the public and private health system perspective, patients treated with the CFRT strategy lost 3.72 QALY and 3.64 LYG compared to HYPOFRT. Thus, CFRT is dominated by HYPOFRT (i.e., HYPOFRT is both more effective and less costly). Base-case results are described in Table 2 and Supplementary Fig. 1 (Supplementary material).

**Table 2:** Base-case results

Brazilian Unified Healthcare System (SUS) – Public health system								
Strategy	Cost (R$)	Incremental costs (R$)	QALYs	Incremental QALYs	LYG	Incremental LYG	ICER (R$)	
							Costs/QALY	Costs/LYG
HYPOFRT	7,008.54		6.50		8.06			
CFRT	7,993.29	984,74	2.78	-3.72	4.42	-3.64	-264.32	-270.83
Health insurance – Private health system								
Strategy	Cost (R$)	Incremental costs (R$)	QALYs	Incremental QALYs	LYG	Incremental LYG	ICER (R$)	
							Costs/QALY	Costs/LYG
HYPOFRT	77,899.27		6.50		8.06			
CFRT	88,594.23	10,694.96	2.78	-3.72	4.42	-3.64	-2870.69	-2941.41

### Sensitivity analysis

**Fig. 2:**
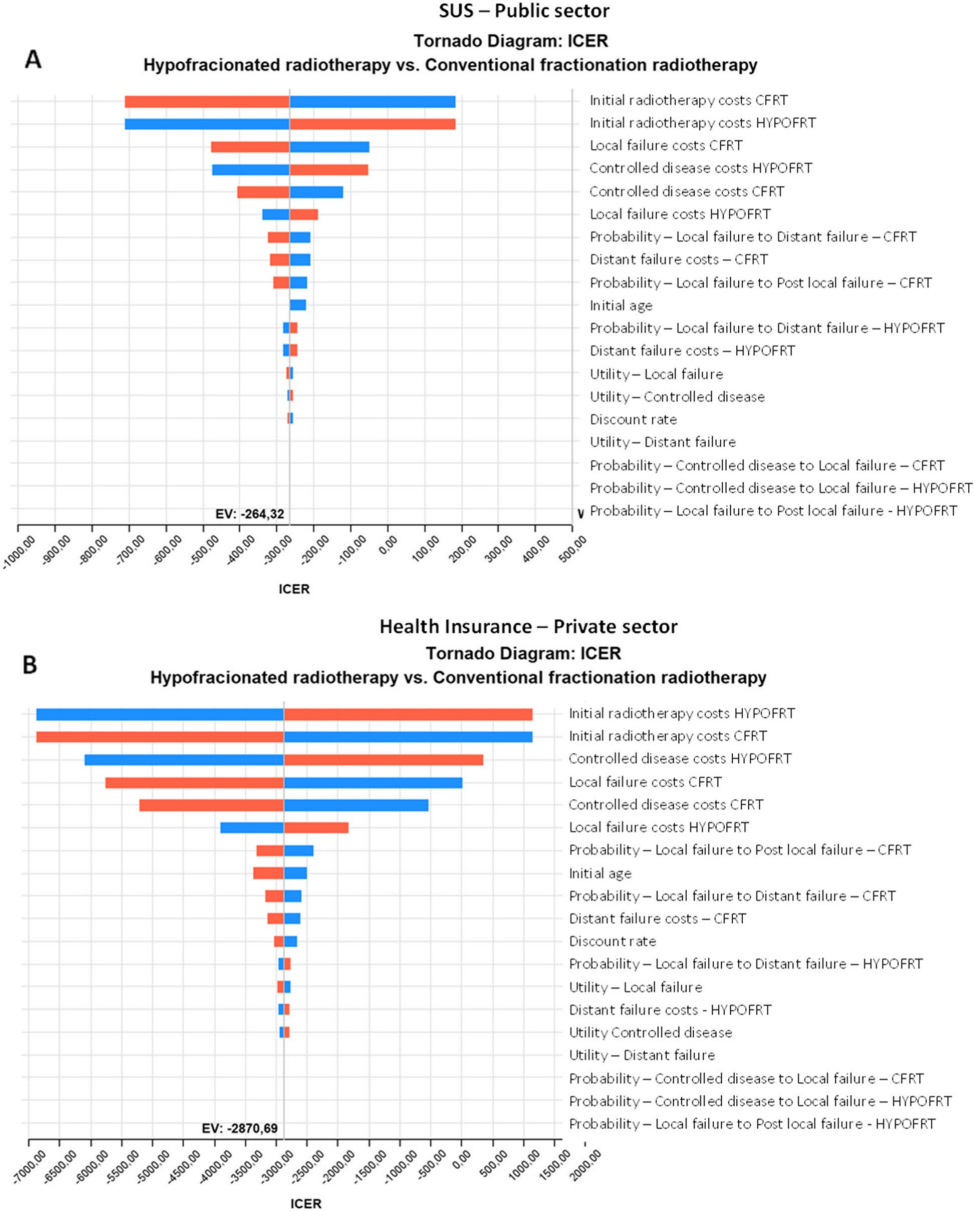
Deterministic sensitivity analyses. Costs – 40% range of the base case; Probabilities and utilities – confidence interval reported at Supplementary Table 2; ICER: Incremental Cost effectiveness analysis

The results for deterministic sensitivity analysis indicated that for both the public health system (Fig. 2A) and private health system (Fig. 2B), the cost-effectiveness was more sensitivity to the initial radiotherapy costs, local failure costs (laryngectomy and palliative chemotherapy), and the costs associated with the controlled disease (follow-up exams and consultations with doctors, dentists, psychologists, nutrition, and speech therapists) (Fig. 2).

To the public health system analysis, when assuming high values for the initial cost of CFRT (R$ 5835.2), the ICER decreased to -R$ 777.82/QALY (-$ 1251.52/ QALY). In a scenario where the initial cost of HYPOFRT was higher (R$ 5835.2), the ICER increased to R$ 183.18/QALY ($ 82.92). If the local failure costs for CFRT increase by 40%, the ICER would decrease to -R$477.43/QALY (-$216.13/QALY). If the costs of controlled disease for HYPOFRT increase by 40%, the ICER would also increase to -R$53.80/QALY (-$24.35/QALY). Accordingly, in a scenario where the controlled disease costs for CFRT were 40% higher, the ICER would decrease to—R$ 408.09 (-$849.31/QALY). Finally, considering local failure costs for HYPOFRT 40% higher, the ICER increases to -R$ 188.07 (-$85.14/QALY).

Concerning the private health system deterministic analysis, when assuming high values for the initial cost of HYPOFRT (R$ 52,354.73), the ICER increased to R$ 1144.39/QALY ($518.06/QALY). Accordingly, if the initial costs of CFRT were higher (R$ 52,354.73), the ICER would decrease to -R$6885.78. In a scenario where the controlled disease costs for HYPOFRT increase by 40%, the ICER would increase to R$ 353.53 ($160.04). Besides, if the local failure costs for CFRT increase by 40%, the ICER would decrease to -R$ 5761.49/QALY (-$2608.19/QALY). If the costs of controlled disease for CFRT increase by 40%, the ICER would also decrease to -R$5209.42/QALY ($2358.27/QALY). Finally, considering that local failure costs for HYPOFRT are 40% higher, the ICER increases to -R$ 1836 ($831.15/QALY).

Thus, for both public and private health systems, the ICERs suggest that for all ranges considered in the deterministic sensitivity analysis, the HYPOFRT strategy is still cost-effective when using the Brazilian cost-effectiveness threshold (R$ 40,000 or $18,107.74). Recommendation from the Brazilian National Technology Incorporation Commission (CONITEC) indicates that the cost-effectiveness threshold for the public health care system in Brazil should be R$40,000/QALY ($ 18,107.74/QALY) and R$ 35,000/LYG ($ 15,844.27/LYG) [32].

**Fig. 3:**
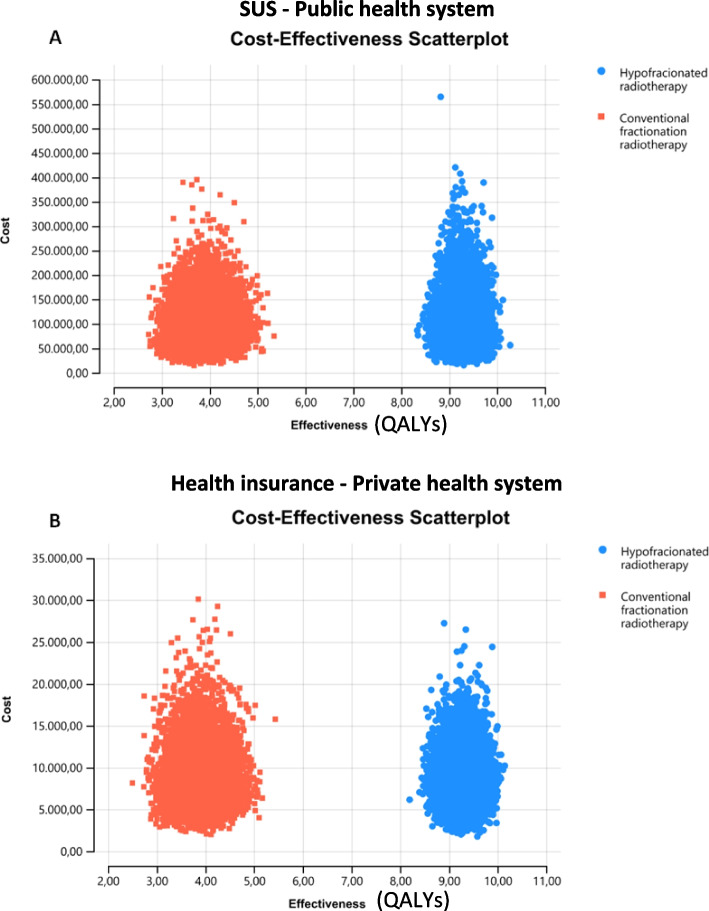
Cost-effectiveness scatter plot

Results from probabilistic sensitivity analysis showed that the HYPOFRT strategy is more effective (generates more QALYs), but it is not more costly than the CFRT strategy for both the public (Fig. 3A) and private health system (Fig. 3B).

**Fig. 4:**
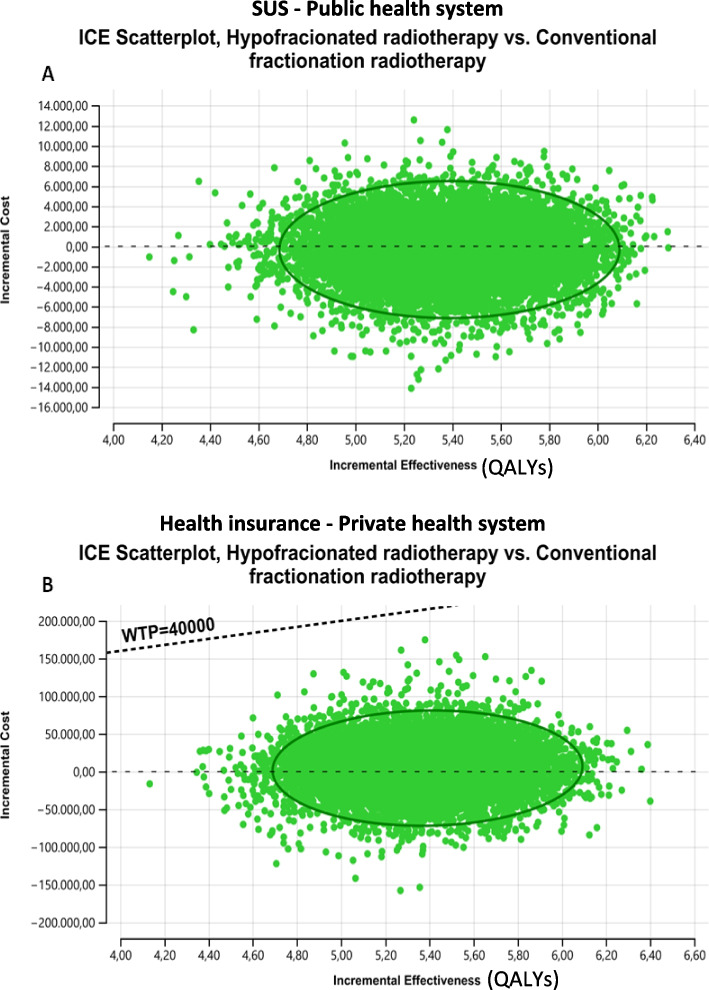
Cost-effectiveness acceptability curve

Besides, the incremental cost-effectiveness scatter plot shows that 100% of the simulated ICERs were cost-effective under the R$ 40,000 ($18,107.74) per QALY gained threshold for both the public (Fig. 4A) and private health system (Fig. 4B).

**Fig. 5:**
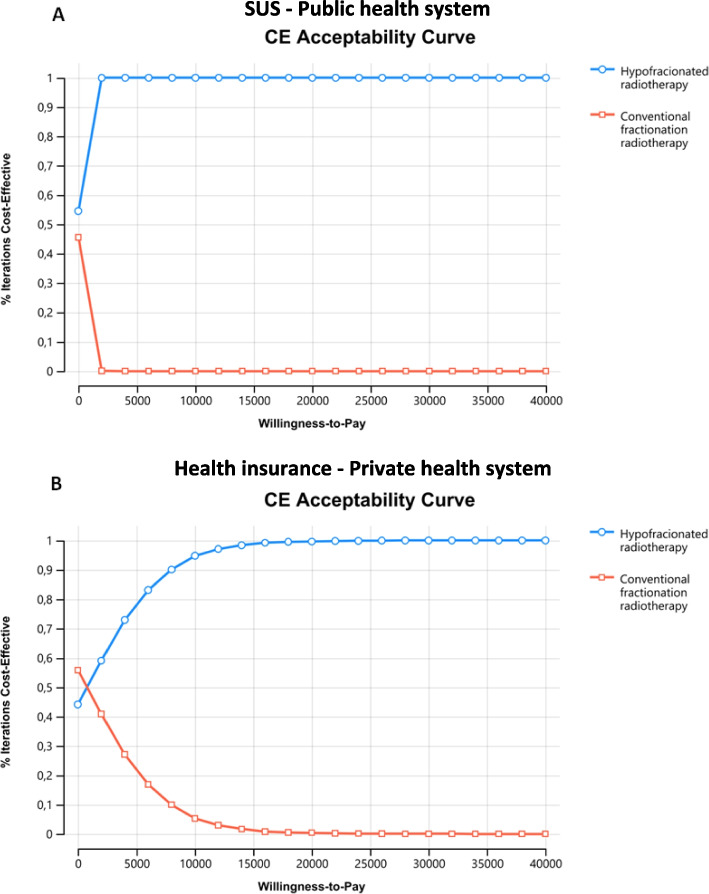
Cost-effectiveness acceptability curve

Accordingly, for the public health system, the cost-effectiveness acceptability curve points out that for a willingness to pay threshold of R$2,000/QALY ($905.39/QALY), there is a probability of 99.99% of HYPOFRT strategy be cost-effective concerning conventional fractionation strategy (Fig. 5A). For the private health system, the cost-effectiveness acceptability curve indicates that for a willingness to pay threshold of R$16,000/QALY ($7243.10/QALY), a probability of 99.99% of HYPOFRT strategy being cost-effective compared to CFRT (Fig. 5B).

**Fig. 6:**
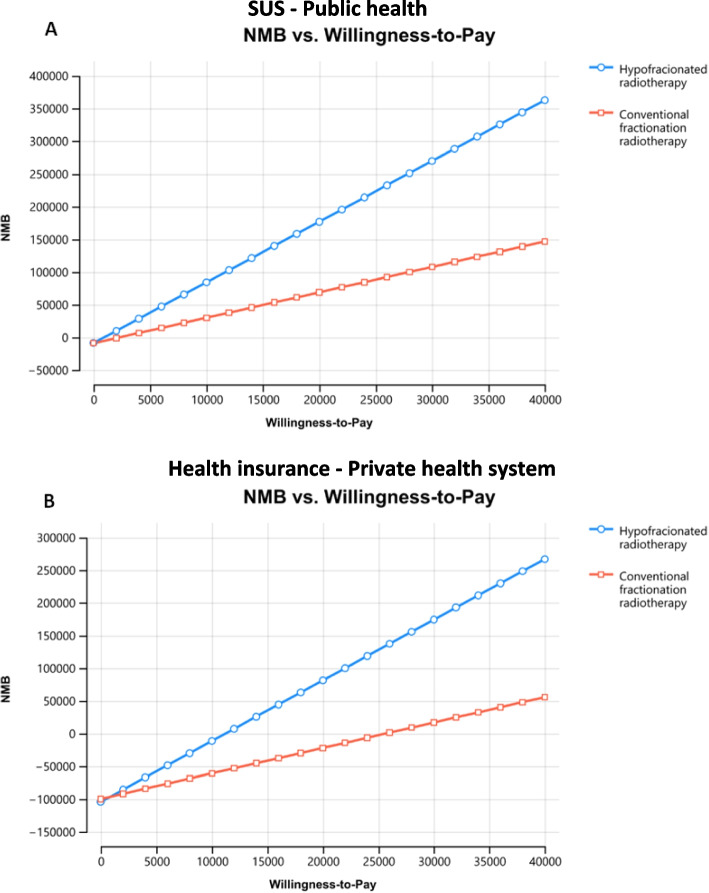
Net Monetary Benefit (NMB) versus Willingness-to-pay

Finally, the probabilistic sensitivity analysis also provided the net monetary benefit (NMB) versus willingness-to-pay analysis, indicating that for the public health system, when considering a willingness-to-pay of R$ 40,000/QALY ($18,107.74/QALY), the NMB is approximately 2.4 times higher for HYPOFRT than for CFRT (Fig. 6A). For the private health system, if we also consider a willingness-to-pay of R$ 40,000/QALY ($18,107.74/QALY), the NMB is approximately 5.2 times higher for HYPOFRT than for CFRT (Fig. 6B).

## Discussion

Our data shows HYPOFRT is cost-effective compared to CFRT for ESGC treated in the Brazilian public and private health systems. In Brazil, the RT reimbursement in the public and private health system is fixed and done equally, independent of the fractionation or technique employed to treat ESGC. The Brazilian reimbursement model confers a unique opportunity to compare these two treatment strategies for cost-effectiveness. The HYPOFRT strategy was more effective and less costly, resulting in a negative ICER of R$ 264.32 ($199.66) per QALY (public health system) and a negative ICER of R$ 2870.69 ($1299.54) per QALY (private health system). The sensitivity analysis also revealed a superiority of HYPOFRT even using the values practiced by the private health system, which are 200% higher than the value reimbursed by the public health system. Considering a willingness-to-pay of 40,000/QALY, the NMB is approximately 2.4 times higher for HYPOFRT than for CFRT using public health system reimbursement values and 5,2 times with private health system values.

This outcome is highly significant from the payer perspective in a country with limited resources to invest in the health system. First, it indicates that more resources are saved as more HYPOFRT is used. Second, the saved resources could be employed to improve the quality of patient network care by readjusting the values or incorporating new technologies to the roll of paid procedures such as Intensity-modulated radiation therapy (IMRT) and/or image-guided radiotherapy (IGRT). IMRT is more time-consuming and expensive than conventional (2DRT) or conformational (3DRT). The Brazilian public reimbursement of radiation therapy procedures has not been readjusted since 2010 [15].

Additionally, IMRT is not yet incorporated as a strategy of radiation therapy for the head and neck in the public setting. This lack of monetary correction associated with the reduced value reimbursed by the public system makes many radiotherapy services not use IMRT to treat their patients. Although conventional radiotherapy (2D or 3DRT) provides high local control rates and low rates of severe complications, this technique has inherent limitations for ESGC [33]. The main problem with these older techniques is the dose delivered to the adjacent normal tissue, such as the common carotid arteries and the thyroid gland [33]. The benefits of dose reduction to the carotid arterial system with IMRT in ESGC are abounding in the literature [33–37]. Retrospective studies have identified an increased risk of carotid artery stenosis and stroke for head and neck patients who receive RT [38]. For instance, a recent analysis of Surveillance, Epidemiology, and End Results (SEER), including ESGC patients treated with EBRT, showed a significant associated risk of developing a fatal stroke to patients treated with surgery [39].

Our premise was that using HYPOFRT could represent an opportunity to save resources and incorporate technology such as IMRT, IGRT, and MRI planning in clinical practice. The outcomes of the deterministic sensitivity analysis reinforce the hypothesis of incorporating technology using as a strategy the HYPOFRT. The sensitivity analysis shows that by increasing the initial cost of HYPOFRT up to R$37,396.24 ($17,297.06), which is the amount value paid by the private health system for IMRT, the HYPOFRT would still be cost-effective compared to CFRT. Thus, the sensitivity analysis using the values of RT reimbursement of the private health system shows that even with a readjusting in the values paid by the public system, HYPOFRT still has remained cost-effective. In light of our results, a reassessment of the cost-effectiveness of IMRT for head and neck cancer in combination with HYPOFRT might be opportune. The cost savings associated with using HYPOFRT instead of CFRT could create the opportunity of investing in supplementary technology such as IMRT, IGRT, and MRT in clinical practice, and future economic analyses are warranted to confirm this hypothesis. It is crucial to note that, in 2017, the Brazilian health technology assessment considered that implementing IMRT for head and neck cancer in the public system was not cost-effective [40]. However, in their evaluation, it was not considered the HYPOFRT strategy.

For the public and private health systems, the acceptability curve and the scatter plot resulted in high precision and trustful outcome with 99.9% and 100% in the Monte-Carlo simulation (10.000 interactions). It is a significant outcome for the payers once it clarifies what intervention to choose. Based on those outcomes appears reasonable to infer that HYPOFRT for ESGC should be considered the standard in Brazil. The LINAC shortage in the country also reinforces this point once HYPOFRT could be used to treat other tumors such as breast, lung, and prostate. However, using the HYPOFRT for these cancer sites also requires technology acquisition, such as IMRT and IGRT, to maintain the therapeutic index [41].

While our investigation shows significant findings, it is relevant to highlight some limitations. First, this study evaluated a cohort of 65-year-old men; women were excluded from the analysis. This choice was made because glottic cancer is more common in men than women. The National Cancer Institute (INCA) estimates that, for each year of the 2020–2022 triennium, 7,650 new cases of laryngeal cancer are diagnosed in Brazil (6,470 in men and 1,180 in women). These values correspond to an estimated risk of 6.20 cases per 100,000 men and 1.06 cases per 100,000 women. About 60% of laryngeal cancer cases start in the glottis, while 35% develop in the supraglottic region. The rest start in the subglottis or more than one area [42]. However, considering that glottic cancer does occur in women, further studies might evaluate if the HYPOFRT strategy is also cost-effective for treating women with ESGC once studies have indicated a significant difference in radiation response between men and women [43, 44], suggesting that gender should be considered when designing treatment protocols [44].

Second, the strategies of carotid sparing were not addressed in the Yamazaki trial, and we could not evaluate the possible impact of radiation on carotid vessels and its economic consequences. It is an essential drawback of this analysis and should be addressed in future trials, which could generate additional advantages of technological incorporation in laryngeal cancer.

Third, our HYPOFRT cost-effectiveness analysis was limited to only early-stage glottic cancer, limiting our findings to other tumors. Fourth, our estimations were developed with probabilities and utilities from other countries due to the lack of data on the Brazilian population. To mitigate this limitation, the face validity of all input data was conducted by consulting different Brazilian experts. Fifth, the hospitalization and patients’ travel costs were not assessed; if they were considered, the HYPOFRT cost would probably be smaller than CFRT because the use of HYPOFRT results in a reduced number of treatment sessions which can contribute to reducing patients’ financial toxicity (fewer travels to receive the treatment) and hospitalization costs.

The present study develops a Markov model that might contribute to designing further modeling studies to evaluate the cost-effectiveness of other cancer sites, radiotherapy modalities, and countries. Markov and partitioned survival models (PSM) are commonly used for the cost-effective analysis of cancer treatments. In this study, we have chosen to use the Markov model due to the lack of individual patient data and the simplicity of modeling the course of disease based on previous Markov models [29, 30]. Moreover, there is no evidence that Markov and PSM lead to different results, and both are recommended as appropriate for cancer treatments [45].

The literature on cost-effectiveness analysis from upper-middle-income countries is limited, but they are essential to acquiring new interventions and technology in a health system with limited budgets. Finally, this analysis is practically relevant because if policy decisions markers choose to implement the cost-effective strategy, it can contribute to improving patients’ quality of life and reducing the waiting lines due to HYPOFRT's reduced number of treatment sessions, which are excellent outcomes for a country with LINACs shortage to treat cancer patients.

## Conclusion

HYPOFRT is cost-effective compared to CFRT for ESGC in the Brazilian public health system. The NMB produced by HYPOFRT could be used to incorporate new technology into the health system, such as IMRT/ VMAT. The probabilistic sensitivity analysis of the outcomes resulted in high precision and low uncertainty, which means that in Brazil, the government should consider the HYPOFRT the standard of care for ESGC.

## Supplementary Information

**Additional file 1.** 

## Data Availability

The main data generated or analyzed during this study are included in this published article [and its supplementary information files]. A more detailed dataset used and analyzed during the current study is available from the corresponding author upon reasonable request.

## Abbreviations

HYPOFRT: Hypofractionated radiotherapy
CFRT: Conventional fractionated radiotherapy
ESGC: Early-stage glottic cancer
ICER: Cost-effectiveness ratio
QALY: Quality-adjusted life-year
LYG: Life years gained
NMB: Net Monetary Benefit
CBHPM: Brazilian Hierarchical Classification of Medical Procedures
SUS: Brazilian Unified Healthcare System
