# Supplementary material for: Cost-effectiveness of hypofractionated versus conventional fractionated radiotherapy for the treatment of men with early glottic cancer: a study in the Brazilian public and private health system
Source: BMC Health Serv Res. 2023 Jun 8;23:592. doi: 10.1186/s12913-023-09397-5 (PMC10249165; doi:10.1186/s12913-023-09397-5)
Supplement: Supplementary file 1 — Additional file 1. [file 12913_2023_9397_MOESM1_ESM.docx]

**Supplementary material**

**Supplementary Table 1** | Checklist for reporting economic evaluations

**Supplementary Table 2 |** Detailed costs per health state and year

**Supplementary Table 3 |** Search strategies

**Supplementary Table 4 |** Inputs used in the Markov Model: probabilities, utilities, and cost

**Supplementary Figure 1 |** Cost and QALYs of both strategies

Supplementary Table 1 – The CHEERS 2022 checklist for reporting economic evaluations

| Section/topic | Item No | Guidance for reporting | Reported  in section |
| --- | --- | --- | --- |
| Title | | | |
| Title | 1 | Identify the study as an economic evaluation and specify the interventions being compared. | Title |
| Abstract | | | |
| Abstract | 2 | Provide a structured summary that highlights context, key methods, results, and alternative analyses. | Abstract |
| Introduction | | | |
| Background and objectives | 3 | Give the context for the study, the study question, and its practical relevance for decision making in policy or practice. | Introduction |
| Methods | | | |
| Health economic analysis plan | 4 | Indicate whether a health economic analysis plan was developed and where available. | Not applicable |
| Study population | 5 | Describe characteristics of the study population (such as age range, demographics, socioeconomic, or clinical characteristics). | Methods section |
| Setting and location | 6 | Provide relevant contextual information that may influence findings. | The first part of the methods |
| Comparators | 7 | Describe the interventions or strategies being compared and why chosen. | Strategies for comparison and model overview |
| Perspective | 8 | State the perspective(s) adopted by the study and why chosen. | The first part of the methods |
| Time horizon | 9 | State the time horizon for the study and why appropriate. | The first part of the methods |
| Discount rate | 10 | Report the discount rate(s) and reason chosen. | The first part of the methods |
| Selection of outcomes | 11 | Describe what outcomes were used as the measure(s) of benefit(s) and harm(s). | The first part of the methods |
| Measurement of outcomes | 12 | Describe how outcomes used to capture benefit(s) and harm(s) were measured. | The first part of the methods |
| Valuation of outcomes | 13 | Describe the population and methods used to measure and value outcomes. | The first part of the methods |
| Measurement and valuation of  resources and costs | 14 | Describe how costs were valued. | Costs section |
| Currency, price date, and conversion | 15 | Report the dates of the estimated resource quantities and unit costs, plus the currency and year of conversion. | Costs section |
| Rationale and description of model | 16 | If modelling is used, describe in detail and why used. Report if the model is publicly available and where it can be accessed. | Declarations – availability of data and materials |
| Analytics and assumptions | 17 | Describe any methods for analysing or statistically transforming data, any extrapolation methods, and approaches for validating any model used. | Methods |
| Characterising heterogeneity | 18 | Describe any methods used for estimating how the results of the study vary for subgroups. | Not applicable |
| Characterising distributional effects | 19 | Describe how impacts are distributed across different individuals or adjustments made to reflect priority populations. | Not applicable |
| Characterising uncertainty | 20 | Describe methods to characterise any sources of uncertainty in the analysis. | Model validation and sensitivity analysis |
| Approach to engagement with  patients and others affected by  the study | 21 | Describe any approaches to engage patients or service recipients, the general public, communities, or stakeholders (such as clinicians or payers) in the design of the study. | Model validation and sensitivity analysis |
| Results | | | |
| Study parameters | 22 | Report all analytic inputs (such as values, ranges, references) including uncertainty or distributional assumptions. | Table 1 |
| Summary of main results | 23 | Report the mean values for the main categories of costs and outcomes of interest and summarise them in the most appropriate overall measure. | Table 2 |
| Effect of uncertainty | 24 | Describe how uncertainty about analytic judgments, inputs, or projections affect findings. Report the effect of choice of discount rate and time horizon, if applicable. | Results |
| Effect of engagement with patients and others affected by the study | 25 | Report on any difference patient/service recipient, general public, community, or stakeholder involvement made to the approach or findings of the study | Costs section |
| Discussion | | | |
| Study findings, limitations,  generalisability, and current  knowledge | 26 | Report key findings, limitations, ethical or equity considerations not captured, and how these could affect patients, policy, or practice. | Discussion |
| Other relevant information | | | |
| Source of funding | 27 |  | Funding section |
| Conflicts of interest | 28 |  | Competing interests’ section |

Supplementary Table 2 – Detailed costs per health state and year

| **Cost of controlled disease** | | | | | | | | | | | | |
| --- | --- | --- | --- | --- | --- | --- | --- | --- | --- | --- | --- | --- |
| **Cost per procedure group (%)** | | **First-year** | | **Second year** | | **Third year** | | **Fourth-year** | | **Fifth year** | | |
|  | **Unit price (R$)** | **Qt** | **Total (R$)** | **Qt** | **Total (R$)** | **Qt** | **Total (R$)** | **Qt** | **Total (R$)** | **Qt** | **Total (R$)** |  |
| **Diagnostic** | | | | | | | | | | | | |
| Chest tomography | 136.41 | 1 | 136.41 | 1 | 136.41 | 1 | 136.41 | 1 | 136.41 | 1 | 136.41 |  |
| Esophagogastroduodenoscopy | 48.16 | 1 | 48.16 | 1 | 48.16 | 1 | 48.16 | 1 | 48.16 | 1 | 48.16 |  |
| Neck tomography | 86.75 | 1 | 86.75 | 1 | 86.75 | 0 | 0 | 0 | 0 | 0 | 0 |  |
| Videolaparoscopy | 95 | 4 | 380 | 4 | 380 | 0 | 0 | 0 | 0 | 0 | 0 |  |
| Chest radiograph | 6.88 | 0 | 0 | 1 | 6.88 | 1 | 6.88 | 1 | 6.88 | 1 | 6.88 |  |
| Glutamic oxaloacetic transaminase (GOT) – liver function | 2.01 | 1 | 2.01 | 0 | 0 | 0 | 0 | 0 | 0 | 0 | 0 |  |
| Glutamic-pyruvic transaminase (GPT) - liver function | 2.01 | 1 | 2.01 | 0 | 0 | 0 | 0 | 0 | 0 | 0 | 0 |  |
| Measurement of total bilirubin and fractions (liver function) | 2.01 | 1 | 2.01 | 0 | 0 | 0 | 0 | 0 | 0 | 0 | 0 |  |
| Creatinine measurement | 1.85 | 1 | 1.85 | 0 | 0 | 0 | 0 | 0 | 0 | 0 | 0 |  |
| Measurement of free thyroxine (free t4) | 11.6 | 1 | 11.6 | 0 | 0 | 0 | 0 | 0 | 0 | 0 | 0 |  |
| Complete blood count | 4.11 | 1 | 4.11 | 0 | 0 | 0 | 0 | 0 | 0 | 0 | 0 |  |
| Electrolytes | 5.55 | 1 | 5.55 | 0 | 0 | 0 | 0 | 0 | 0 | 0 | 0 |  |
| Measurement of thyroid-stimulating hormone (TSH) | 8.96 | 1 | 8.96 | 1 | 8.96 | 1 | 8.96 | 1 | 8.96 | 1 | 8.96 |  |
| **Clinical procedures** | | | | | | | | | | | | |
| Medical consultation | 10 | 4 | 40.00 | 4 | 40.00 | 2 | 20.00 | 2 | 20.00 | 2 | 20.00 |  |
| Phonoaudiological consultation | 6.3 | 9 | 56.7 | 0 | 0 |  | 0 |  | 0 |  | 0 |  |
| Nutritionist consultation | 6.3 | 1 | 6.3 | 0 | 0 |  | 0 |  | 0 |  | 0 |  |
| Dental Consultation | 6.3 | 1 | 6.3 | 0 | 0 |  | 0 |  | 0 |  | 0 |  |
| Psychological consultation | 6.3 | 4 | 25.2 | 0 | 0 |  | 0 |  | 0 |  | 0 |  |
| **Sum per health state per year** | | **823.92** | | **707.16** | | **220.41** | | **220.41** | | **220.41** | | |
| **Cost of local failure** | | | | | | | | | | | | |
| **Cost per procedure group** | | **First-year** | | **Second year** | | **Third year** | | **Fourth-year** | | **Fifth year** | | |
|  | **Unitary price** | **Qt** | **Total** | **Qt** | **Total** | **Qt** | **Total** | **Qt** | **Total** | **Qt** | **Total** |  |
| **Surgical procedures** | | | | | | | | | | | | |
| Total laryngectomy with neck dissection | 980.31 | 1 | 980.31 | 0 | 0 | 0 | 0 | 0 | 0 | 0 | 0 |  |
| **Diagnostic** | | | | | | | | | | | | |
| Follow-up exams per year* | - | 1 | 689.42 | 1 | 667.16 | 1 | 200.41 | 1 | 200.41 | 1 | 200.41 |  |
| **Clinical procedures** | | | | | | | | | | | | |
| Follow-up consultations per year* | - | 19 | 134.5 | 4 | 40.00 | 2 | 20.00 | 2 | 20.00 | 2 | 20.00 |  |
| **Sum per health state per year** | | **1804.23** | | **707.16** | | **220.41** | | **220.41** | | **220.41** | | |
| **Distant failure** | | | | | | | | | | | | |
| **Cost per procedure group** | | **First-year** | | **Second year** | | **Third year** | | **Fourth-year** | | **Fifth year** | | |
|  | **Unitary price** | **Qt** | **Total** | **Qt** | **Total** | **Qt** | **Total** | **Qt** | **Total** | **Qt** | **Total** |  |
| **Clinical procedures** | | | | | | | | | | | | |
| Chemotherapy of advanced head and neck squamous cell carcinoma - (palliative chemotherapy) | 800.00 | 6 | 4800.00 | 0 | 0 | 0 | 0 | 0 | 0 | 0 | 0- |  |
| **Diagnostic** | | | | | | | | | | | | |
| Follow-up exams per year** | - | 1 | 466.26 | 1 | 444.00 | 1 | 200.41 | 1 | 200.41 | 1 | 200.41 |  |
| **Clinical procedures** | | | | | | | | | | | | |
| Follow-up consultations per year* | - | 19 | 134.50 | 4 | 40.00 | 2 | 20.00 | 2 | 20.00 | 2 | 20.00 |  |
| **Sum per health state per year** | |  | 5400.76 |  | 484.00 |  | 220.41 |  | 220.41 |  | 220.41 |  |

* The costs of follow-up exams per year were the sum of diagnostics procedures per year discriminated in the controlled disease part, and the costs of follow-up consultations per year were the sum of clinical procedures per year that were also discriminated in the controlled disease part.

** The costs of follow-up exams per year were the sum of diagnostics procedures that were discriminated in controlled disease part less the costs of the tomographs

Supplementary Table 3 - Search strategies

| Search | Query | Results |
| --- | --- | --- |
| [#1] | "early glottic carcinoma"[Title/Abstract] OR "early glottic cancer"[Title/Abstract] OR "early-stage glottic carcinoma"[Title/Abstract] OR "early-stage glottic cancer"[Title/Abstract] OR "t1 glottic cancer"[Title/Abstract] OR "t1 glottic carcinoma"[Title/Abstract] OR "T1N0M0"[Title/Abstract] | 1612 |
| [#2] | "hypofractionated"[Title/Abstract] OR "hypofractionated schedule"[Title/Abstract] OR "hypofractionated radiation regimen"[Title/Abstract] OR "hypofractionated radiotherapy"[Title/Abstract] OR "hypofractionated radiation therapy"[Title/Abstract] OR "radiation fraction size"[Title/Abstract] | 4280 |
| [#3] | "conventional radiotherapy"[Title/Abstract] OR "conventional schedule"[Title/Abstract] OR "conventional radiation therapy"[Title/Abstract] OR "conventional fractionation radiotherapy"[Title/Abstract] OR "conventional fractionated radiotherapy"[Title/Abstract] OR "conventional fractionation scheme"[Title/Abstract] OR "conventional fractionation schedule"[Title/Abstract] OR "conventional fractionation regimen"[Title/Abstract] OR "radiation fraction size"[Title/Abstract] | 3058 |
| [#4] | [#1] AND [#2] AND [#3]  ("early glottic carcinoma"[Title/Abstract] OR "early glottic cancer"[Title/Abstract] OR "early stage glottic carcinoma"[Title/Abstract] OR "early stage glottic cancer"[Title/Abstract] OR "t1 glottic cancer"[Title/Abstract] OR "t1 glottic carcinoma"[Title/Abstract] OR "T1N0M0"[Title/Abstract]) AND ("hypofractionated"[Title/Abstract] OR "hypofractionated schedule"[Title/Abstract] OR "hypofractionated radiation regimen"[Title/Abstract] OR "hypofractionated radiotherapy"[Title/Abstract] OR "hypofractionated radiation therapy"[Title/Abstract] OR "radiation fraction size"[Title/Abstract]) AND ("conventional radiotherapy"[Title/Abstract] OR "conventional schedule"[Title/Abstract] OR "conventional radiation therapy"[Title/Abstract] OR "conventional fractionation radiotherapy"[Title/Abstract] OR "conventional fractionated radiotherapy"[Title/Abstract] OR "conventional fractionation scheme"[Title/Abstract] OR "conventional fractionation schedule"[Title/Abstract] OR "conventional fractionation regimen"[Title/Abstract] OR "radiation fraction size"[Title/Abstract]) | 6 |
| [#5] | Filters: Randomized Controlled Trial | 2 |

Supplementary Table 4 – Inputs used in the Markov Model: probabilities, utilities, and cost

| Variable | Min | Value | Max | Source |
| --- | --- | --- | --- | --- |
| Probabilities |  |  |  |  |
| p_CD_CD_HYPOFRT |  |  |  |  |
| Year 1 | 0.953766 | 0.966258 | 0.97875 | (8) |
| Year 2 | 0.944563 | 0.957055 | 0.969547 | (8) |
| Year 3 | 0.92918 | 0.941672 | 0.954164 | (8) |
| Year 4 | 0.92918 | 0.941672 | 0.954164 | (8) |
| Year 5 | 0.917646 | 0.930138 | 0.94263 | (8) |
| p_CD_CD_CFRT |  |  |  |  |
| Year 1 | 0.887014 | 0.94907 | 1 | (8) |
| Year 2 | 0.838677 | 0.900733 | 0.96279 | (8) |
| Year 3 | 0.792803 | 0.854859 | 0.916915 | (8) |
| Year 4 | 0.749265 | 0.811321 | 0.873377 | (8) |
| Year 5 | 0.707944 | 0.770000 | 0.832056 | (8) |
| p_LF_DF |  |  |  |  |
| Year 1 | 0 | 0.008851 | 0.020849 | (8) |
| Year 2 | 0.005626 | 0.017624 | 0.029621 | (8) |
| Year 3 | 0.014321 | 0.026319 | 0.038316 | (8) |
| Year 4 | 0.022939 | 0.034937 | 0.046934 | (8) |
| Year 5 | 0.031481 | 0.043478 | 0.055476 | (8) |
| p_LF_Death |  |  |  |  |
| Year 1 | 0 | 0.025243 | 0.058075 | (8) |
| Year 2 | 0.049848 | 0.049848 | 0.082681 | (8) |
| Year 3 | 0.073832 | 0.073832 | 0.106665 | (8) |
| Year 4 | 0.097211 | 0.097211 | 0.130044 | (8) |
| Year 5 | 0.12 | 0.12 | 0.152833 | (8) |
| p_DF_Death |  |  |  |  |
| Year 1 | 0.461536 | 0.601893 | 0.742249 | (8) |
| Year 2 | 0.701154 | 0.841511 | 0.981867 | (8) |
| Year 3 | 0.796548 | 0.936904 | 1.077261 | (8) |
| Year 4 | 0.834525 | 0.974881 | 1.115237 | (8) |
| Year 5 | 0.849644 | 0.99 | 1.130356 | (8) |
| P_CD_Death | 0.03 | 0.02 | 0.04 | (28) |
| Utility |  |  |  |  |
| Controlled disease | 0.83 | 0.85 | 0.86 | (22) |
| Local failure | 0.51 | 0.57 | 0.63 | (23) |
| Distant failure | 0.42 | 0.427 | 0.43 | (24) |
| Costs (R$) Public Health System |  |  |  |  |
| HYPOFRT_and_CVRT_initial_costs | 2500.8 | 4168 | 5835.2 | (25) |
| CD |  |  |  |  |
| Year 1 | 494.35 | 823.92 | 1.153.49 | (25) |
| Year 2 | 424.30 | 707.16 | 990.02 |  |
| Year 3 | 132.25 | 220.41 | 308.57 |  |
| Year 4 | 132.25 | 220.41 | 308.57 |  |
| Year 5 | 132.25 | 220.41 | 308.57 |  |
| LF |  |  |  |  |
| Year 1 | 1082.54 | 1804.23 | 2525.92 | (25) |
| Year 2 | 424.30 | 707.16 | 990.02 |  |
| Year 3 | 132.25 | 220.41 | 308.57 |  |
| Year 4 | 132.25 | 220.41 | 308.57 |  |
| Year 5 | 132.25 | 220.41 | 308.57 |  |
| DF |  |  |  |  |
| Year 1 | 3374.35 | 5623.92 | 7873.49 | (25) |
| Year 2 | 424.30 | 707.16 | 990.02 |  |
| Year 3 | 132.25 | 220.41 | 308.57 |  |
| Year 4 | 132.25 | 220.41 | 308.57 |  |
| Year 5 | 132.25 | 220.41 | 308.57 |  |
| Costs (R$) Private Health System |  |  |  |  |
| HYPOFRT_and_CVRT_initial_costs |  | 37396.24 |  |  |
| CD |  |  |  |  |
| Year 1 | 8.767.08 | 14.611.79 | 20456.51 | (26) |
| Year 2 | 8.351.44 | 13.919.07 | 19486.70 |  |
| Year 3 | 1738.61 | 2897.68 | 4056.76 |  |
| Year 4 | 1738.61 | 2897.68 | 4056.76 |  |
| Year 5 | 1738.61 | 2897.68 | 4056.76 |  |
| LF |  |  |  |  |
| Year 1 | 12503.20 | 20.838.67 | 29.174.13 | (26) |
| Year 2 | 8351.44 | 13.919.07 | 19.486.70 |  |
| Year 3 | 1738.61 | 2897.68 | 4056.76 |  |
| Year 4 | 1738.61 | 2897.68 | 4056.76 |  |
| Year 5 | 1738.61 | 2897.68 | 4056.76 |  |
| DF |  |  |  |  |
| Year 1 | 14527.08 | 24211.79 | 33896.51 | (26) |
| Year 2 | 8351.44 | 13919.07 | 19486.70 |  |
| Year 3 | 1738.61 | 2897.68 | 4056.76 |  |
| Year 4 | 1738.61 | 2897.68 | 4056.76 |  |
| Year 5 | 1738.61 | 2897.68 | 4056.76 |  |

Abbreviations: CD, controlled disease; LD, local failure; PLF, Post-local failure; DF, distant failure; CBHPM, Hierarchical Brazilian Classification of Medical Procedures.


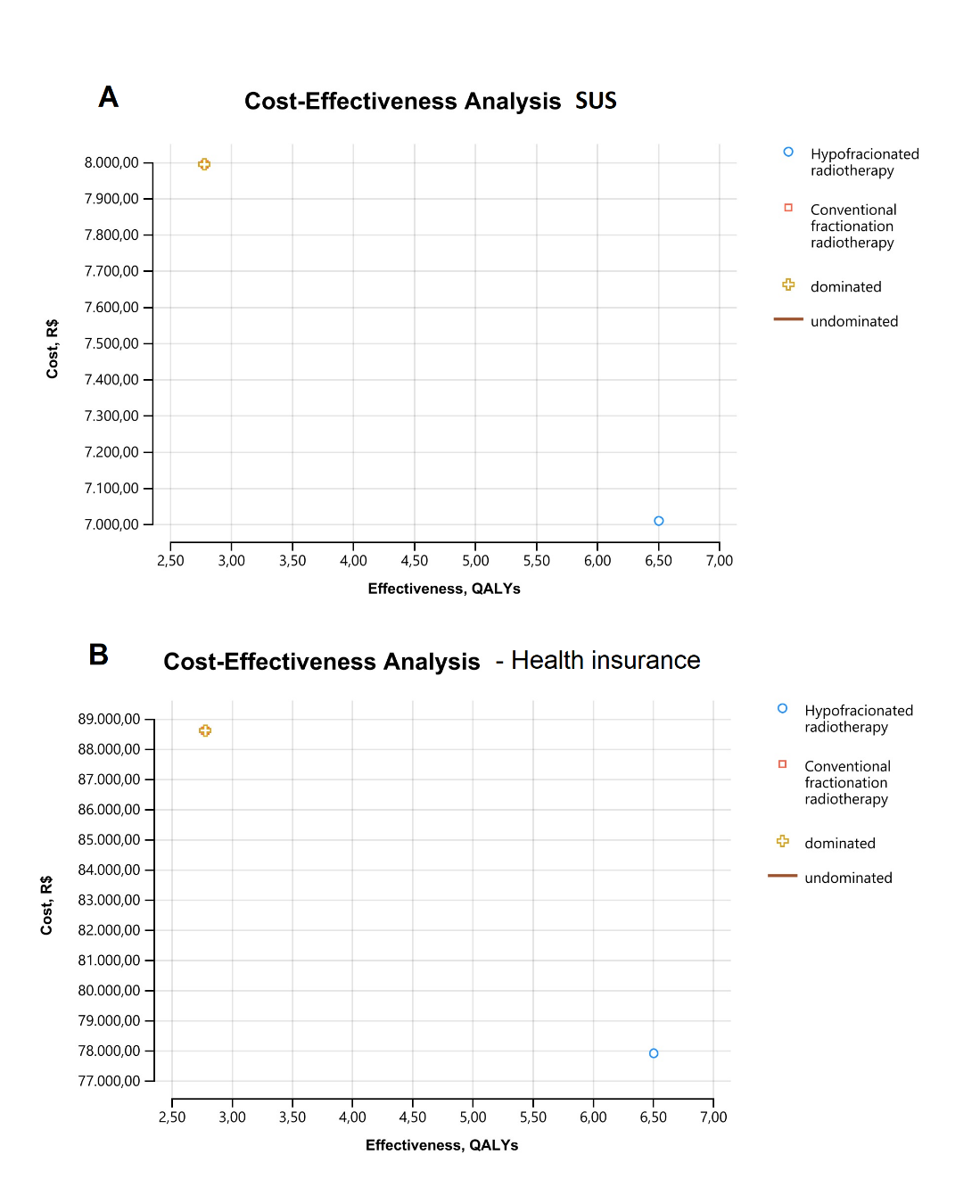
Supplementary Figure 1 – Cost and QALYs of both strategies
